# P-1053. Invasive Fusariosis: 5-Year Update from a Single-Center Experience in the Voriconazole Era

**DOI:** 10.1093/ofid/ofae631.1242

**Published:** 2025-01-29

**Authors:** Victor Kovac, Lindsey R Baden, Ann E Woolley

**Affiliations:** Brigham and Women's Hospital, Boston, Massachusetts; Brigham and Women's Hospital, Boston, Massachusetts; Brigham and Women's Hospital, Boston, Massachusetts

## Abstract

**Background:**

Invasive fusariosis is a rare but aggressive infection seen in immunocompromised patients that is often multidrug resistant. The optimal therapeutic approach in the presence of elevated MICs to standard antifungal treatment remains unknown and careful monitoring of local epidemiology of this infection is needed. We performed a retrospective review of invasive fusarium infections to help guide management at our center.

**Methods:**

We identified all cases of invasive Fusarium infection between January 2019 and April 2024. We collected patient characteristics, including clinical presentation, therapy, and 12-week outcomes, as well as organism speciation and susceptibility testing.

**Results:**

Seven patients were diagnosed with either proven (6, 86%) or probable (1, 14%) invasive fusariosis. The median age was 60 years and six (86%) patients were male. Four (57%) had hematologic malignancies, of which 3 (75%) received stem cell transplant, 1 (14%) idiopathic neutropenia, 1 (14%) severe burns, and 1 (14%) received solid organ transplant. Four (57%) had prolonged preceding neutropenia. Cutaneous lesions were the most common presentation (5, 71%). The four cases in neutropenic hosts had cutaneous lesions with presumed or confirmed disseminated disease involving the sinuses or lung. Three (43%) received combination therapy with voriconazole and terbinafine, 2 (29%) received posaconazole, 1 (14%) received amphotericin, and 1 (14%) received voriconazole. *Fusarium solani* complex was the most commonly isolated species (2, 29%). Species identification was not available in 3 cases. Susceptibility testing was available in 5 (71%) cases and MICs to voriconazole were ≥16 µg/mL and to terbinafine were ≥2. All 4 cases treated with voriconazole had elevated MICs and yet exhibited a partial (2, 50%) or complete clinical response (2, 50%) to treatment. 12-week survival was seen in 4 (57%) of cases, with one patient lost to follow-up.

**Conclusion:**

Mortality to invasive fusariosis is high but remains similar at our center compared to the 2002-2014 period. Diagnosis remains challenging and tissue biopsy is critical. Despite elevated MICs, clinical responses to voriconazole with or without the addition of terbinafine are observed.

**Disclosures:**

**All Authors**: No reported disclosures

